# Expression of *FcFT1*, a *FLOWERING LOCUS T-*like gene, is regulated by light and associated with inflorescence differentiation in fig (*Ficus carica* L.)

**DOI:** 10.1186/1471-2229-13-216

**Published:** 2013-12-16

**Authors:** Hidetoshi Ikegami, Hitoshi Nogata, Yoshiaki Inoue, Shuichi Himeno, Hiroshi Yakushiji, Chiharu Hirata, Keita Hirashima, Masashi Mori, Mitsuo Awamura, Takao Nakahara

**Affiliations:** 1 Fukuoka Agricultural Research Center, 587 Yoshiki, Chikushino, Fukuoka 818-8549, Japan; 2 Fukuoka Prefectural Control Station for Pests, 423 Yoshiki, Chikushino, Fukuoka 818-0004, Japan; 3 Fukuoka Agricultural Research Center Buzen Station, 2-4-1 Nishiizumi, Yukuhashi, Fukuoka 824-0038, Japan; 4 Grape and Persimmon Research Station, National Institute of Fruit Tree Science, National Agriculture and Food Research Organization, NARO, Akitsu 301-2, Higashi Hiroshima, Hiroshima 739-2494, Japan; 5 Ishikawa Prefectural University, 921-8836 Suematsu, Nonoichi, Ishikawa 834-1213, Japan; 6 Fukuoka Agricultural Research Center Yame Station, 3266-1 Honbun, Kuroki, Yame, Fukuoka 834-1213, Japan

**Keywords:** Bearing habit, Floral differentiation, *Flowering locus T*, Light regulation

## Abstract

**Background:**

Because the floral induction occurs in many plants when specific environmental conditions are satisfied, most plants bloom and bear fruit during the same season each year. In fig, by contrast, the time interval during which inflorescence (flower bud, fruit) differentiation occurs corresponds to the shoot elongation period. Fig trees thus differ from many species in their reproductive growth characteristics. To date, however, the molecular mechanisms underlying this unorthodox physiology of floral induction and fruit setting in fig trees have not been elucidated.

**Results:**

We isolated a *FLOWERING LOCUS T* (*FT*)*-*like gene from fig and examined its function, characteristics, and expression patterns. The isolated gene, *F. carica FT* (*FcFT1*), is single copy in fig and shows the highest similarity at the amino acid level (93.1%) to apple *MdFT2*. We sequenced its upstream region (1,644 bp) and identified many light-responsive elements. *FcFT1* was mainly expressed in leaves and induced early flowering in transgenic tobacco, suggesting that *FcFT1* is a fig *FT* ortholog. Real-time reverse-transcription PCR analysis revealed that *FcFT1* mRNA expression occurred only in leaves at the lower nodes, the early fruit setting positions. mRNA levels remained a constant for approximately 5 months from spring to autumn, corresponding almost exactly to the inflorescence differentiation season. Diurnal variation analysis revealed that *FcFT1* mRNA expression increased under relative long-day and short-day conditions, but not under continuous darkness.

**Conclusion:**

These results suggest that *FcFT1* activation is regulated by light conditions and may contribute to fig’s unique fruit-setting characteristics.

## Background

Fig (*Ficus carica* L.) is a deciduous, subtropical, semiarboreal fruit tree belonging to *Ficus*, a genus of 600 to 1,900 species in the family of Moraceae [1,2]. It is considered one of the earliest domesticated plants of the Neolithic Revolution [3]. The fig tree has unique fruit-bearing characteristics and is traditionally associated with abundance and fertility.

**Figure 1:**
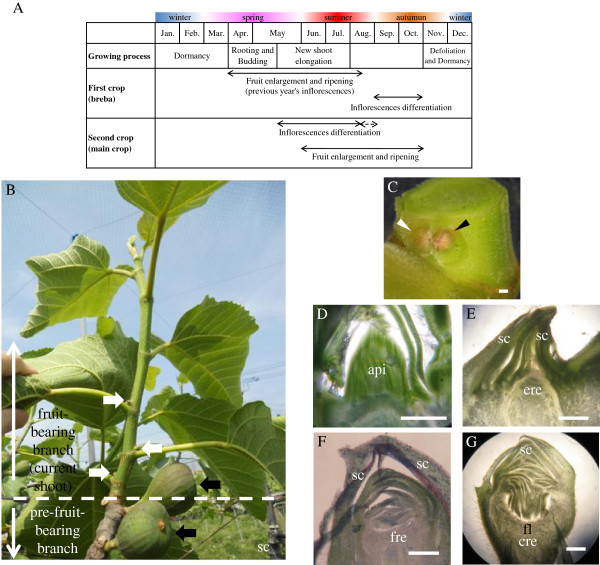
**Bearing style and flower bud differentiation in *Ficus carica *L. (A)** Fig fruit (‘Houraishi’) production in Japan. The broken arrow indicates the period when inflorescences differentiate but most resulting fruits drop or decay because of winter low temperature. **(B)** First-crop fruits differentiate at nodes near the tip of 2-year-old pre-fruit-bearing branches (black arrows) and second-crop fruits differentiate at nodes near the base of 1-year-old fruit-bearing branches (white arrows). Inflorescences differentiate sequentially from lower nodes as a 1-year-old fruit-bearing branch develops. **(C)** Exterior appearance of leaf bud (left white arrowhead) and flower bud (right black arrowhead) at axillary position. **(D)** Histological sections of apical bud meristem (api). No inflorescences were observed before bud flushing. **(E)** Histological sections of a flower bud with an early receptacle (ere) and scales (sc). **(F)** Histological sections of a flower bud with a flat receptacle (fre) and scales (sc). **(G)** Histological sections of a flower bud with a curved receptacle (cre) surface on which floret (fl) differentiation begins. Bar = 500 μm.

The details of fig fruit (inflorescences) growth and development have been described in many previous studies [4]. When terminal and axillary buds of the pre-fruit-bearing branches (2-year-old shoots) elongate in spring, the cover scales abscise and the apical meristem develops into a shoot that produces leaves and new inflorescences [5]. The inflorescence differentiation process occurs sequentially, starting from the lower nodes, which first bear fruit, and progressing toward the higher ones. Because fig fruits are composed of an enlarged receptacle with hundreds to thousands of florets inside (a syconium), it is reasonable to assume that differentiation of inflorescences and fruits occurs simultaneously [6]. Succession in maturation towards the distal end continues as long as environmental conditions are favorable [7]. In autumn, fruits differentiate at nodes near the tip of fruit-bearing branches (1-year-old shoots) and then, become dormant. Because of low temperatures, they do not ripen during winter. Fruit hypertrophy begins the following spring with ripening during summer (Figure 1A, 1B).

Fig trees consequently produce two fruit crops per year, one in early summer (first crop) and the other in early autumn (second crop) [8]. However, this does not mean that the inflorescence differentiation event occurs only twice annually. Inflorescence differentiation actually takes place throughout the shoot elongation period [7]. The first and second crops only appear to have differentiated at independent times because the first crop’s differentiation and developmental seasons are separated by low-temperature-induced winter dormant period (Figure 1A).

Because floral induction occurs in many plants when specific environmental conditions, such as day length, temperature, autonomous factors, or some combination thereof, are satisfied, it usually occurs annually at a certain time. For example, in *Arabidopsis*, flowering occurs frequently in response to long-day conditions during spring or summer [9]. In rice, flowering (termed heading) is promoted by short-day conditions during summer or autumn [10]. In poplar, a woody perennial, cold temperatures promote reproductive onset during winter [11]. By contrast, fig reproductive growth continues for most of the growing season, a long period extending from spring through autumn. Fig is, thus, presumed to follow a floral induction model that differs from those of most other plant species. To date, however, the molecular mechanisms underlying this unorthodox physiology of inflorescences differentiation and fruit-setting in fig trees have not been identified.

Decades of studies have revealed many genes that control floral induction in various plants, including the model organism *Arabidopsis*[12-17]. Among these genes, *FLOWERING LOCUS T* (*FT*) and its associated family are well known as integrative genes that induce flowering, because they encode possible florigen components [18-21] and serve as crossover points for photoperiodic and vernalization pathways [20]. As a first step in elucidating the mechanisms that underlie and control flowering in fig trees, it is therefore important to investigate the function and features of the fig *FT* homolog.

In this study, we cloned and characterized the first known *FT*-like gene from fig, *FcFT1*. In addition, we confirmed that *FcFT1* can enhance floral induction and that its expression mode is unique. *FcFT1* may therefore be responsible for the unique flowering and fruit-setting characteristics of fig trees.

## Methods

### Inflorescence investigation and microscopic analysis

To confirm that inflorescences differentiate only in the current year of bearing, apical buds and inflorescences (bud crowns) [4] were studied by light microscopy after their collection in March and May 2011 from a ‘Houraishi’ cultivar grown in an open field at the Fukuoka Agricultural Research Center (FARC), Yukuhashi, Japan. Sections (0.04-0.08 mm thick) were cut with a TH desktop hand microtome (Kenis, Osaka, Japan) and stained with 0.05% (w/v) toluidine blue. Images were acquired under a BH-2 light microscope (Olympus, Tokyo, Japan) equipped with a Power Shot A590 Image Stabilizer (IS) digital camera (Cannon, Tokyo, Japan). The number of fruits set in a ‘Houraishi’ adult tree was also investigated from late May to late July to assess whether fig has a continuous bearing habit. Inflorescences larger than 2 mm were considered fruit. We counted the total number of fruits set on eight branches.

### Gene and promoter cloning and phylogenetic analysis

Cloning of the fig *FT* gene was performed using a 3′/5′rapid amplification of cDNA ends (RACE) strategy. First, we obtained an *FT*-like fragment by genomic PCR to design gene-specific primers. Degenerate primers for genomic PCR were designed from conserved regions of *FT*-family genes in other plant species. Genomic DNA was extracted from mature leaves of young rooted cuttings of ‘Houraishi’ from FARC [22,23] using a DNeasy Plant Mini kit (Qiagen, Hilden, Germany). RACE PCR was performed using a GeneRacer kit (Invitrogen, Carlsbad, CA, USA). We used the primer sequences shown in Additional file 1: Table S1. The generated 5′ and 3′ fragments were aligned, and a complete cDNA sequence was identified. The complete *FcFT1* coding region was obtained by PCR from cDNA and genomic DNA using KOD Plus polymerase (Toyobo, Osaka, Japan). The amplified sequences were cloned into a pCR-Script Amp SK (+) vector (Agilent Technologies, La Jolla, CA, USA) and fully sequenced. Sequencing was performed using a Big-Dye Terminator Cycle Sequencing kit on an ABI Prism 310 sequencer (Applied Biosystems, Sunnyvale, CA, USA).

Genome walking to isolate the 5′ upstream sequence flanking the *FcFT1* coding region was performed by the Straight Walk method [24] using a Straight Walk kit (Bex, Tokyo, Japan) in accordance with the supplier’s instructions. Cis-elements of the 1,644-bp 5′ upstream sequence were predicted using the PLACE Signal Scan Search program (http://www.dna.affrc.go.jp/PLACE/signalscan.html).

Phylogenetic and molecular evolutionary analyses were conducted using the GENETYX software package (ver. 8.0; Genetyx, Tokyo, Japan). To generate a phylogenetic tree, predicted proteins and FT proteins of other plant species were aligned with a multi-sequence alignment program using the default parameters. To estimate evolutionary distances and construct the tree, the proportion of amino acid differences was computed using the amino acid neighbor-joining method.

### DNA gel blot analyses

**Figure 2:**
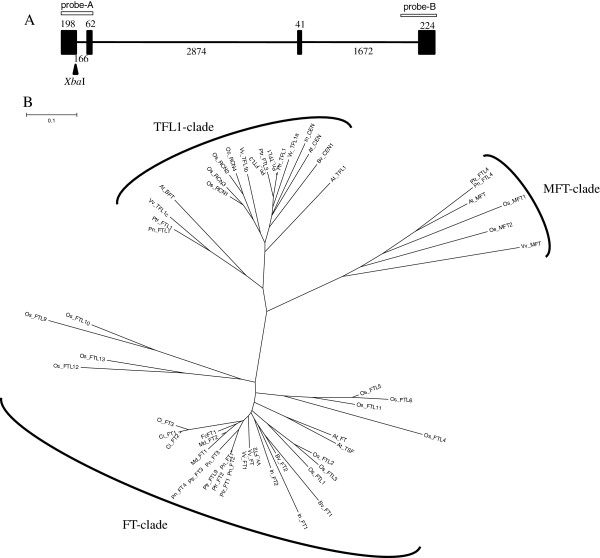
**Similarities between the deduced protein sequence of the *FT-like *gene of *Ficus carica*, *FcFT1*, and other *FT *homologs.** Panel **A**, genomic structure of *FcFT1*. White bars indicate the hybridization positions of *FcFT1* probes, and closed arrowheads show enzyme restriction sites (*Xba*I). Panel **B**, the phylogenetic tree of the *FT* family based on amino acid sequences. Accession numbers of sequences used are as follows: *FcFT1* (AB457620) from *Ficus carica*; *AtFT* (*FT*) (AB027504), *AtTSF* (*TSF*) (NP_193770), *AtTFL1* (*TFL1*) (NM_120465), *AtBFT* (*BFT*) (NP_201010), (*AtCEN* (*ATC*) (AB024715), *AtMFT* (*MFT*) (AEE29676) from *Arabidopsis thaliana*; *BvFT1* (HM448910), *BvFT2* (HM448912), *BvCEN1* (HM448914) from *Beta vulgaris*, *CiFT* (AB027456), *CiFT2* (AB301934), and *CiFT3* (AB301935) from *Citrus unshiu*; *InFT1* (ABW73562), *InFT2* (ABW73563) from *Ipomoea nil*, *MdFT1* (AB161112) and *MdFT2* (FJ555224) from *Malus* × *domestica*; *OsFTL1* (LOC_Os01g11940), *OsFTL2* (LOC_Os06g06320), *OsFTL3* (LOC_Os06g06300), *OsFTL4* (LOC_Os09g33850), *OsFTL5* (LOC_Os02g39064), *OsFTL6* (LOC_Os04g41130), *OsFTL9* (LOC_Os01g54490), *OsFTL10* (LOC_Os05g44180), *OsFTL11* (LOC_Os11g18870), *OsFTL12* (LOC_Os06g35940), *OsFTL13* (LOC_Os02g13830), *OsMFT1* (LOC_Os06g30370), *OsMFT2* (LOC_Os01g02120), *OsRCN1* (LOC_Os11g05470), *OsRCN2* (LOC_Os02g32950), *OsRCN3* (LOC_Os12g05590), *OsRCN4* (LOC_Os04g33570) from *Oryza sativa*; *PnFT1* (BAD01612), *PnFT2* (BAD01561), *PnFT3* (BAD02371), *PnFT4* (BAG12904), *PnFTL1* (BAD27481), *PnFTL3* (BAD22601), *PnFTL4* (BAD22677), *PnTFL1* (BAD22599) from *Populus nigra*; *PtrFT1* (XP_002316173), *PtrFT2* (XP_002334306), *PtrFT3*(XP_002311264), *PtrFTL1* (XP_002321903), *PtrFTL3* (XP_002312811), *PtrFTL4* (ABC26020), *PtrFTL9* (XP_002334492), *PtrTFL1*(XP_002328260) from *Populus trichocarpa*; and *VvFT* (ABF56526), *VvFT1* (ABI99465), *VvFT2* (ABL98120), *VvMFT* (ABI99469), *VvMFT2* (XP_002281565), *VvTFL1a* (ABI99466), *VvTFL1b* (ABI99467), and *VvTFL1c* (ABI99468) from *Vitis vinifera*.

Three fig cultivars, ‘Houraishi’, ‘Masui Dauphine’, and ‘Toyomitsuhime’, were used for DNA gel blot analyses. Analyses were conducted as described by Brown [25]. Two probes ­probe A and probe B­ were designed to target the region from the 1^st^ exon to the 1^st^ intron and the region from the 3^rd^ intron to the 4^th^ exon, respectively (Figure 2A). The restriction enzymes *Xba*I and *Hind*III were used to digest DNA.

### Generation and phenotypic analysis of tobacco transgenic lines

The complete coding sequence of *FcFT1* with *Xba*I and *Sac*I adapter sites was cloned into a pE2113 vector under the control of PR1a [26] or El2-35S-Ω [27] promoters. PR1a and El2-35S-Ω allowed for the examination of weakly and strongly expressing transformants, respectively. These constructs were introduced into the *Agrobacterium tumefaciens* EHA105 strain using a modified cold-shock method [28]. The resultant *Agrobacterium* strains were used to transform sterile seedlings of *Nicotiana tabacum* ‘Samsun NN’ using the leaf-disc method [29]. Transgenic plants were selected on MS medium containing 3% sucrose and 0.3% Gelrite supplemented with 50 μg l^-1^ hygromycin. Plants were grown under a 16-h light/8-h dark photoperiod. The successful introduction of the *FcFT1* gene to produce transformants was confirmed by genomic PCR using *FcFT1*-FL1 and *FcFT1*-RL1 primers (Additional file 1: Table S1).

For phenotypic characterization, we first collected T2 seeds from *FcFT1* transformants. Seeds collected from the respective lines were grown aseptically in MS, and potted in horticultural soil after 21 days. We evaluated the following parameters for all transgenic plants: number of days from sowing to flowering, number of leaves, and plant height 70 days after sowing. Traits of wild-type and transgenic lines were compared using the Tukey-Kramer method.

### Analysis of *FcFT1* expression in fig trees

Plant materials were collected from different organs for real-time reverse-transcription (RT)-PCR analyses. *FcFT1* mRNA expression levels were investigated at the fruit stage I in leaves, stems, receptacles, pericarps, and florets of 3-year-old rooted cuttings of ‘Houraishi’ grown in an artificial weather chamber. To evaluate changes in *FcFT1* mRNA spatial and seasonal expression, we sampled upper halves of three leaves from bearing branches of 22- to 24-year-old adult ‘Houraishi’ trees growing at FARC. For the spatial analysis, leaves were sampled at each node from shoots elongated to the 10^th^ node. Gene expression levels were compared using Tukey’s test (P < 0.05). Seasonal sampling was conducted from 13:00 to 14:00 once a month from March to October 2011. To examine diurnal variance, samples were collected every 4 h from 3-year-old seedlings growing in an artificial weather chamber (photon flux density, 150 μmol m^-2^ s^-1^; temperature, 25°C). The seedlings were first maintained under a 12-h light/12-h dark photoperiod (12L/12D) for 3 days, and then under relatively long-day light conditions (16L/8D), relatively short-day conditions (8L/16D), or continuous darkness for 3 days (DD).

Real-time RT-PCR reactions were carried out as follows: total RNA was extracted from each sample using an RNeasy Plant Mini kit (Qiagen), with Fruit-mate for RNA Purification (Takara, Shiga, Japan) used in the case of fruit parts [30], and treated with DNase I (Takara). Analyses were conducted according to the real-time RT-PCR kit protocol (One-Step SYBR PrimeScript RT-PCR Kit II Perfect Real Time; Takara) using an ABI Prism 7500 Fast Real-Time PCR system (Life Technologies, Carlsbad, CA, USA). For each sample, 25 ng of total RNA was used. The β-actin gene (DDBJ ID: AY487315) was used as a control [23]. Primer sequences for this experiment are shown in Additional file 1: Table S1. Thermal cycling conditions were as follows: 5 min at 42°C, 10 s at 95°C, and 45 cycles of 5 s at 95°C and 34 s at 68°C.

## Results

### Inflorescence development in fig

Microscopic analyses in March showed that differentiation of flat receptacles had not occurred at apical buds of the previous year. By May 15, differentiation of flat receptacles had taken place at the lower-node inflorescences on bearing branches (the current shoots), but not at the upper nodes. Florets, which were characterized by masses of circular primordia, had emerged by late May (Figure 1C-G). Fruit bearing was observed from May, when inflorescences first differentiated, until July or even later (Additional file 2: Figure S1).

### Identification of an *FT* homolog in fig

A full-length cDNA was cloned using the RACE techniques from an RNA sample of fig leaves. Comparisons of the deduced protein sequence with *FT* and *FT*-like sequences from other species as well as the results of the sequence alignment indicated that the cloned sequence was an *FT* homolog. We designated the gene as *FcFT1* (GenBank accession no. AB457620). Genomic structural analysis of *FcFT1* revealed that the gene was 5,175-bp long and had four exons and three introns, similar to other genes in the *FT* family (Figure 2A). The second and third introns, comprising 2.9 kbp and 1.7 kbp, respectively, contained many mononucleotide runs.

Comparison of complete amino acid sequences indicated 74.5% identity of *FcFT1* with *FT* (DDBJ ID: AB027504) and 92.5% identity with *MdFT2* (DDBJ ID: FJ555224). A tree generated by phylogenetic analysis of the amino acid sequences contained three major clades supporting TFL1, MFT and *FT* subfamilies (Figure 2B). The *FcFT1* sequence also possessed all the characteristic features of the *FT* protein subfamily, including conservation of Try 85 and Gln 140 that are critical for *FT* activity [31,32].

In DNA gel blot analyses, single bands were detected upon hybridization of the probes A or B with *Hind*III-digested DNA. One to two bands were detected in analyses involving probes A or B and the *Xba*I digestion (Additional file 3: Figure S2).

### Analysis of the *FcFT1* upstream sequence

Two bands were acquired twice in a row using the Straight Walk kit. A 1,672-bp sequence was finally identified as the *FcFT1* 5′ upstream region (Additional file 4: Figure S3). We used PLACE to search for motifs in the *FcFT1* upstream sequence, and found many cis-acting regulatory elements for photoresponsiveness and tissue-specific gene expression. These regulatory elements included DOFCOREZM [33,34], CACTFTPPCA1 [35], and CAATBOX1 [36] (Additional file 5: Table S2).

### Functional analyses of *FcFT1* in transgenic tobacco

**Figure 3:**
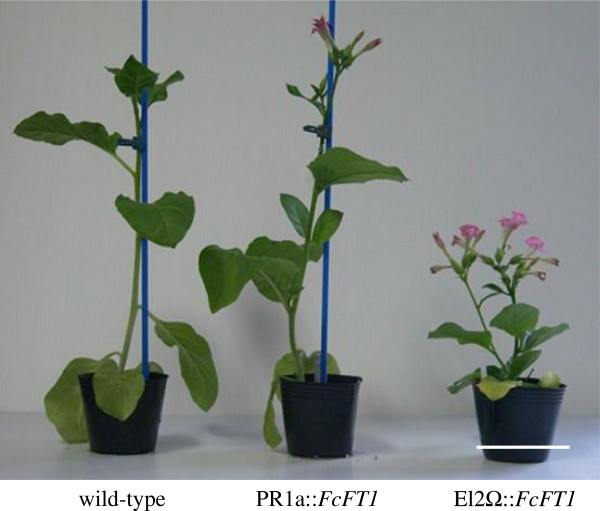
**Flowering phenotypes of transgenic tobacco lines expressing the cDNA of the *FT*-like gene, *FcFT1*, from *Ficus carica *L. El2Ω::*FcFT1 *transgenic plant (T2) (right), PR1a::*FcFT1 *transgenic plant (T1) (center), and wild-type (left) plant in pots 49 days after sowing.** Scale bar = 9 cm.

We conducted tobacco transformation experiments to functionally analyze the *FcFT1* gene product. We obtained numerous transgenic lines from two independent transformation experiments, one with a construct containing the PR1a promoter and the other with a construct containing the El2-35S-Ω promoter. We selected three independent lines for phenotypic analyses, and confirmed the introduction and expression of *FcFT1* in these lines. The PR1a::*FcFT1* and El2-35S-Ω::*FcFT1* transgenic lines bloomed approximately 17 and 23 days earlier, respectively than the wild type. The transgenic lines had fewer leaves per plant and a shorter plant height than did the wild-type line (Table 1; Figure 3).

**Table 1:** **Phenotypes of transgenic tobacco lines expressing constructs containing the *FT*-like gene, *FcFT1*, from *Ficus carica *** **L**

**Genotype**	**n**	**Day to flowering**^**§**^	**Leaf number**^**#**^	**Plant height**
Wild type	15	62.6 ± 0.9^a^	22.0 ± 1.5^a^	53.9 ± 1.3^a^
PR1a::*FcFT1* no.3	14	45.5 ± 0.9^b^	6.1 ± 0.4^b^	30.4 ± 1.2^b^
PR1a::*FcFT1* no.6	15	44.1 ± 1.4^b^	6.6 ± 0.9^b^	19.2 ± 1.4^c^
E12Ω::*FcFT1* no.6	10	39.6 ± 1.3^c^	6.8 ± 0.4^b^	30.0 ± 0.9^b^

^§^Days from sowing to petal opening (± SE). ^#^Number of leaves on 70-day-old plants (±SE). ^a^, ^b^, and ^c^: significantly different from wild-type Samsun NN (SNN) at 70 days of age (Tukey-Kramer method, *P* < 0.01). All plants (T2 generation) were first grown on MS medium and then transferred to pots containing horticultural soil.

### Expression pattern of *FcFT1* mRNA in fig trees

**Figure 4:**
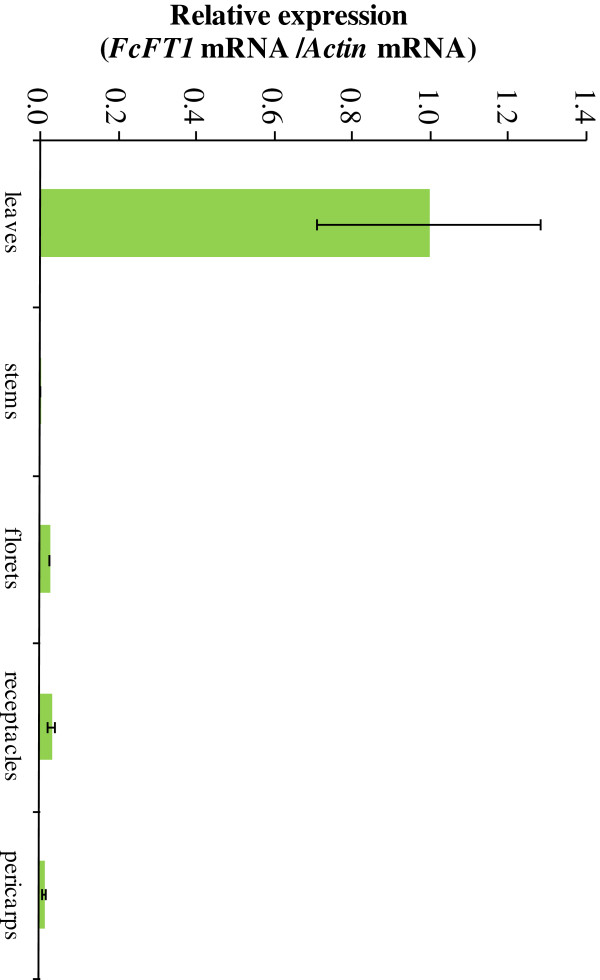
**Real-time RT-PCR analysis of expression of the *FT*-like gene, *FcFT1*, from *Ficus carica *L. in fig trees.** The bar graph shows relative expression of *FcFT1* normalized to *Actin*. Leaves, stems, florets, receptacles, and pericarps (at the young fruit period) were collected from a 3-year-old ‘Houraishi’ fig tree. Error bars show SE (*n* = 3). Error bars for stems and florets are subsumed by symbols.

**Figure 5:**
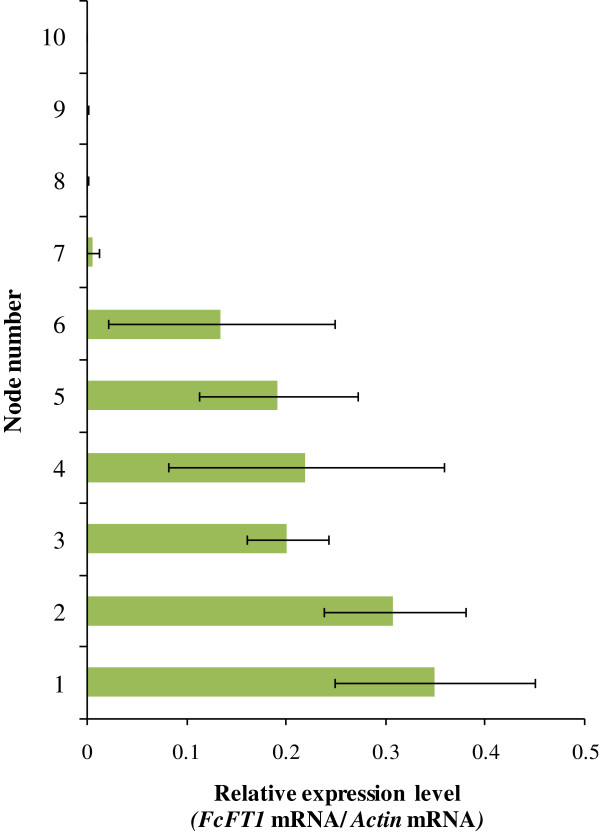
**Expression distribution of mRNA of *FcFT1*, the *FT*-like gene from *Ficus carica *L., from each node leaf on fruit-bearing branches of fig (‘Houraishi’ cultivar).** Node numbering is from the basal node. *FcFT1* mRNA above a threshold level was observed in older nodes that bear fruit earlier (corresponding to the 1^st^ through 6^th^ nodes), while little or no expression was observed in younger nodes that bear fruit later (corresponding to 7^th^ through 10^th^ nodes). Error bars for 8^th^ through 10^th^ nodes are subsumed by the symbols.

We investigated expressions levels of *FcFT1* in each plant organ. *FcFT1* mRNA expression levels were highest in leaves, with expression barely detectable in other organs (Figure 4). In the spatial expression analysis of leaves from each node of current shoots (Figure 5), a higher *FcFT1* mRNA level was observed at the 1^st^ to 6^th^ nodes than at the 7^th^ to 10^th^ nodes. We noticed a negative correlation between node height and observed expression level, but this trend was not statistically significant.

**Figure 6:**
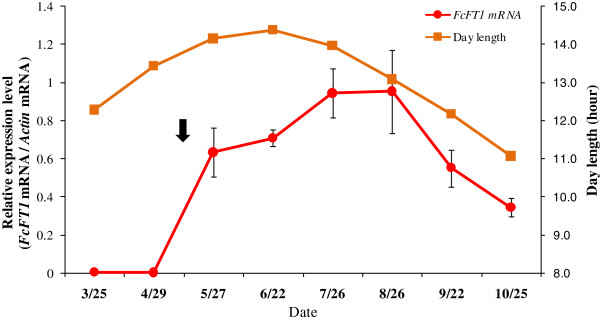
**mRNA expression analyses of *FcFT1*, the *FT*-like gene from *Ficus carica *L., in fig (‘Houraishi’ cultivar) over the 2011 season using real-time RT-PCR.** Each point represents the average of values of the 5^th^ node leaf positions derived from three biological replicates (22- to 24-year-old trees). During seasonal variations, expression levels rapidly increased in May, and continued increasing until August. They remained elevated for as long as 5 months (black arrow). Expression levels then decreased until October, when leaves yellowed. Error bars show SE (*n* = 3). Error bars for March and April are subsumed by the symbols.

**Figure 7:**
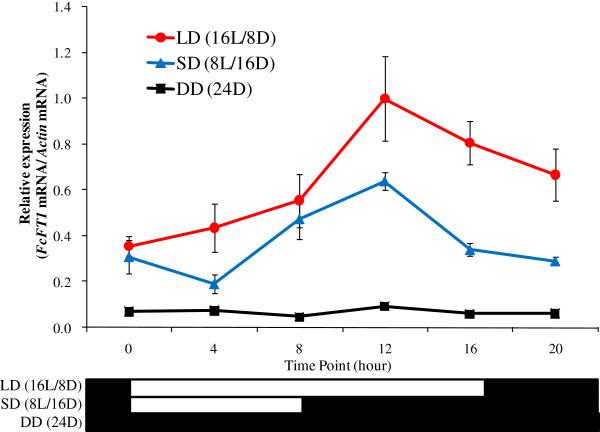
**Diurnal expression pattern of mRNA of *FcFT1*, the *FT*-like gene from *Ficus carica *L., in fig (‘Houraishi’ cultivar) under various photoperiods: 16-h light/8-h dark, LD (16L/8D); 8-h light and 16-h dark, SD (8L/16D); and continuous darkness, DD.** Black boxes indicate darkness; white boxes indicate light. Leaf portions from the upper halves of mature leaves were used for the analyses. Leaves were collected from three independent clones and analyzed at different time points. Error bars show SE (*n* = 3). Error bars for DD (24D) are subsumed by the symbols.

With respect to the seasonal variations, expression levels increased rapidly in May; they continued rising until August, and then decreased until October. These levels remained elevated for as long as 5 months. The highest expression levels were in July and August (Figure 6). We also examined changes in diurnal expression pattern, and detected increased expression under the 16L/8D and 8L/16D photoperiods that peaked 12 h after the start of light illumination. In contrast, expression remained at static low levels under DD (Figure 7).

## Discussion

In many fruit plants, including apple, grape, and persimmon, flower buds (inflorescences) differentiate not in the current year of bearing, but in the previous year [37-40]. In apple, for example, floral primordia appear in summer, with the final formation of flower parts observed in spring. The floral development cycle often lasts 9–10 months [38]. Although the fig inflorescence growth process was investigated by Kimura and Hishiya (1951) [4], no direct evidence has been found to rule out differentiation in the year prior to fruiting. In this study, we found no inflorescences in apical bud meristems before bud flushing (Figure 1D). We were able to confirm that new inflorescences differentiated only after elongation of current shoots (Figure 1E,F,G). In addition, continuous fruit bearing was seen after the first differentiations. These observations demonstrate that generation of new fig inflorescences and floral transitioning occur only after May in the year of fruit bearing, and that floral induction continues even later (Additional file 2: Figure S1).

Our study is the first to report cloning of an *FT* homolog in the genus *Ficus*. Among all known *FT* sequences, *FcFT1* showed the highest identity at the amino acid level with apple *MdFT2*[41]. Based on the DNA gel blot analysis, *FcFT1* exists in a single copy in the fig genome, as single bands were detected with one exception: probe A hybridization combined with *Xba*I digestion, where an *Xba*I recognition site existed in the probe sequence. In that case, the size of the smaller band of ‘Masui Dauphine’ differed from that of ‘Houraishi’ and ‘Toyomitsuhime’, although the number of bands was the same for all three cultivars. This result suggests that some varietal polymorphisms exist in the *FcFT1* flanking region. Because no differences were observed in *FcFT1* cDNA sequences or fruit-bearing styles among these three cultivars, these polymorphisms are assumed to exist in non-coding regions.

The number of days from sowing to flowering, number of leaves, and plant height were reduced in both PR1a::*FcFT1* and El2-35S-Ω::*FcFT1* transgenic lines compared with the wild type (Table 1; Figure 3). In fact, *FcFT1* transgenic tobacco produced small buds, even under Petri dish culture conditions, and showed solid and stable early flowering over subsequent generations. This result provides clear evidence that *FcFT1* has a flower promoting function similar to that of *FT* genes in *Arabidopsis* and other plants [14,42].

*FcFT1* expression levels in leaf tissue were more than 30 times higher than levels detected in stems and fruit (Figure 4), and many mesophyll-specific expression motifs, such as CACTFTPPCA1, were identified in the *FcFT1* promoter sequence (Additional file 1: Table S1). *FcFT1* is therefore presumed to be functional mainly in the leaf, like the *FT* gene of *Arabidopsis*[43]. However, *MdFT2*, which is most similar to *FcFT1*, is expressed mainly in the reproductive organs [37].

As mentioned previously*,* fig inflorescences differentiate from lower to upper nodes. *FcFT1* mRNA levels were higher in lower, older nodes with some inflorescences (1^st^ to 6^th^ nodes) than in upper, younger nodes (7^th^ to 10^th^ nodes) (Figure 5). This result suggests a possible correlation between inflorescence differentiation and *FcFT1* expression. We note, however, that fruit-bearing and *FcFT1*-expressing nodes do not correspond completely, as *FcFT1* expression was detected even in basal nodes that usually bear no fruits in fig. A spatial gradient expression pattern for the *FT* gene has also been reported in tomato [44]. Because the vegetative growth stage is advanced in the lower parts of shoots, degree of vegetative growth may be a regulating factor for *FcFT1* expression.

*FcFT1* mRNA levels increased in May, soon after leaf emergence, and remained constant until October (Figure 6). ‘Houraishi’, at the experimental site, has the ability to differentiate inflorescences and bear fruit over a long time period (Additional file 2: Figure S1). The continuous *FcFT1* expression thus corresponds to this fruit-bearing trend. The fact that the first clear receptacles differentiate at the same time as *FcFT1* expression levels begin to increase, in mid-May, also supports a relationship between *FcFT1* expression and flowering and fruiting (Figure 6).

*FcFT1* was activated above a fixed level under both relatively long-day and short-day conditions, whereas no *FcFT1* activation occurred under continuous darkness (Figure 7). This result suggests that *FcFT1* activation is light mediated. Because expression levels increased immediately upon exposure to light and were higher under a greater light volume, it is likely that *FcFT1* activation is directly influenced by light with higher light levels more favorable for *FcFT1* activation (Figure 7; Additional file 5: TableS2). In addition, the possibility exists that *FcFT1* has lost photoperiodic responsiveness: its diurnal activation pattern is consistent with its seasonally stable expression pattern, whereas day-length changes from season to season (Figure 7). It is not known why the expression level peaks 12 h after dawn. All of these behaviors may serve as clues for the further elucidation of the *FcFT1* light-mediated regulation mechanism.

Many studies on the relationship between light conditions and fig fruit bearing have been published. Matsuura and Araki (1995) reported that inflorescence differentiation and growth could not reach stage I (about 2 mm, the same size as a leaf bud) above the 12^th^ nodes at 75% shading. They also reported that as shading rates increased, a larger number of inflorescences ceased growth before becoming fruit, more fruit yellowed and dropped, and more fruit failed to set above the shoots’ 5^th^ nodes [45]. Teragishi et al. (1998) reported that 8.5-klx light and 14-h day length conditions during the seedling period increased the number of fruit borne, especially below the 5^th^ nodes [46]. These data support the hypothesis that *FcFT1* expression activated by light is indispensable to fruit bearing, including inflorescence differentiation. However, Teragishi et al. (1998) also found that no differences in the fruit-bearing rates between 10 h and 14 h day-light conditions during seedling growth [47]. These data imply that a light volume greater than the fixed amount has little effect on the number of fruit borne. Levels of photosynthesis and the resulting-assimilation products are considered important for fig fruit bearing [5,45,46]. This observation may be linked to the fact that both photosynthesis and *FcFT1* activation are regulated by the same factor.

Taken together, our data strongly suggest that *FcFT1* is a key gene in fig floral induction. In previous studies, large quantities of *FT* transcripts were observed for only 1 or 2 months annually in poplar (*PtFT1* and *PtFT2*) [48-50] and citrus (*CiFT1*, *CiFT2*, *CiFT3*) trees [51]. Although *MdFT1* in apple trees shows a relatively long-term stable expression pattern, it still has an expression peak in July [41]. *FT* and *Hd3a* coordinated by temperature and day length conditions, as described in Background section, are also activated only during specific seasons in *Arabidopsis*[52] and rice [53]. Considering the difference in expression patterns between *FcFT1* and *FT* genes in other species, the distinctive flowering and fruit-bearing characteristics of fig are likely due to the long-term stable expression of *FcFT1.*

To our knowledge, no description has appeared of an *FT* ortholog of a wild-type species that is impervious to variations in photoperiod conditions. In contrast, many such situations have been reported in various mutant lines [54]. In the *Arabidopsis phyB* mutant, the CONSTANS (CO) protein is maintained at a high level, which leads to the promotion of *FT* expression [54,55]. Similar mutants have also been identified in rice and soybean [56-59]. A further example may be the *Arabidopsis* quintuple mutant *cdf1-R cdf2-1 cdf3-1 cdf5-1*, which escaped from the repression of *CO* transcription by the CYCLING DOF FACTORS (CDF) family and induced *FT* mRNA [60].

In *Arabidopsis*, *CO* mRNAs are translated into CO proteins through the light-induced relief of CDFs, with CO proteins stabilized by light through phytochromes and other molecules to induce *FT* gene expression [5,61,62]. We thus hypothesize that malfunction of these factors in the light-dependent pathway in fig causes the apparent light activation of *FcFT1* expression by canceling the repression of *CO* transcription or CO activity. We have identified several CO, phytochrome, and CDF genes in fig (unpublished data); to test our hypothesis, we need to examine the relationship between these genes and *FcFT1*. Any such information uncovered regarding *FcFT1* regulation would prove valuable in terms of providing a novel floral physiology system.

## Conclusions

We isolated an *FT* homolog, *FcFT1*, from *F. carica* and studied its function in transgenic plants. We examined its spatial, seasonal, and diurnal expression patterns in fig, and investigated its association with floral induction (inflorescence differentiation). Our data suggest that *FcFT1*’s unique expression features are responsible for the distinctive flowering and fruit-bearing characteristics of fig, and imply that it plays an important role in fig floral induction. The number of functional *FT*-like genes present in fig, aside from *FcFT1*, is still unknown, however. If other *FT* genes are identified in the future, we will need to also consider their relationship to fig flowering and fruit-bearing characteristics. Nevertheless, the information regarding *FcFT1* obtained in this study should advance understanding of the unique floral transition mechanism of fig trees.

## Competing interests

The authors declare that they have no competing interests.

## Authors’ contributions

HI carried out the molecular genetic studies and drafted the manuscript. HN, YI, SH, and MA cultivated the fig trees and participated in the fruit setting investigation. HY participated in the study design and helped to draft the manuscript. CH and KH contributed to sequencing and sequence alignment. MM and TN were involved in manuscript revision. All authors read and approved the final manuscript.

## Supplementary Material

Additional file 1: Table S1 Primer sequences used in this study. Click here for file

Additional file 2: Figure S1 Number of fruit set on eight bearing branches of an adult ‘Houraishi’ fig tree from late May to late July of 2011. Click here for file

Additional file 3: Figure S2 Southern blot analyses of the *FT*-like gene, *FcFT1*, from *Ficus carica* L. in fig cultivars. Fig genomic DNA was digested with *Xba*I and *Hind*III. Two *FcFT1* genomic DNA fragments were labeled with ^32^P and used as probes. Hybridization and washing were performed under highly stringent conditions as described by Brown (2001). Panel A, digestion with *Xba*I. Panel B, digestion with *Hind*III. Fig cultivars used were as follows: HO, ‘Houraishi’; MA, ‘Masui Dauphine’; TO, ‘Toyomitsuhime’; M, 1 kb ladder marker. Accession numbers: probe-A (AB594722), probe-B (AB594723). Click here for file

Additional file 4: Figure S3 Promoter sequence of the *FT*-like gene, *FcFT1*, from *Ficus carica* L. The 1,644-bp genomic DNA fragment flanking the 5′ end of the gene contains several putative regulatory elements including an underlined TATA-box. Click here for file

Additional file 5: Table S2 Cis-element sequences identified in the 5′ upstream region of the *FT*-like gene *FcFT1,* from *Ficus carica* L. Click here for file
