# Supplementary material for: Expression of FcFT1, a FLOWERING LOCUS T-like gene, is regulated by light and associated with inflorescence differentiation in fig (Ficus carica L.)
Source: BMC Plant Biol. 2013 Dec 16;13:216. doi: 10.1186/1471-2229-13-216 (PMC3878838; doi:10.1186/1471-2229-13-216)
Supplement: Additional file 1: Table S1 — Primer sequences used in this study. [file 1471-2229-13-216-S1.doc]

**Additional file 1: Table S1** Primer sequences used in this study.
