# Supplementary figures and images for: Expression of FcFT1, a FLOWERING LOCUS T-like gene, is regulated by light and associated with inflorescence differentiation in fig (Ficus carica L.)
Source: BMC Plant Biol. 2013 Dec 16;13:216. doi: 10.1186/1471-2229-13-216 (PMC3878838; doi:10.1186/1471-2229-13-216)

## Slide 1
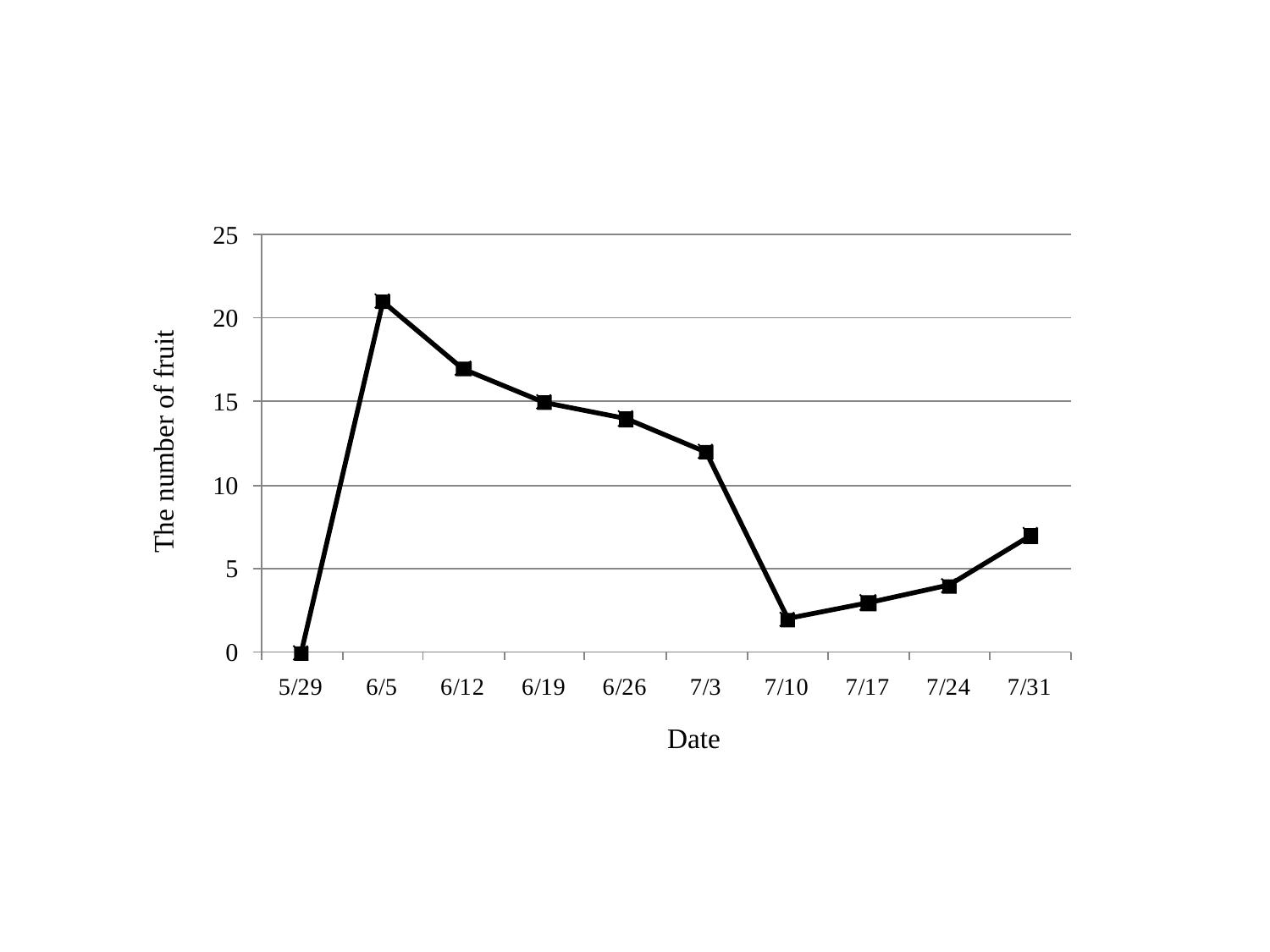

The number of fruit
Date

Supplement: Additional file 2: Figure S1 — Number of fruit set on eight bearing branches of an adult ‘Houraishi’ fig tree from late May to late July of 2011. [file 1471-2229-13-216-S2.pptx]

## Slide 1
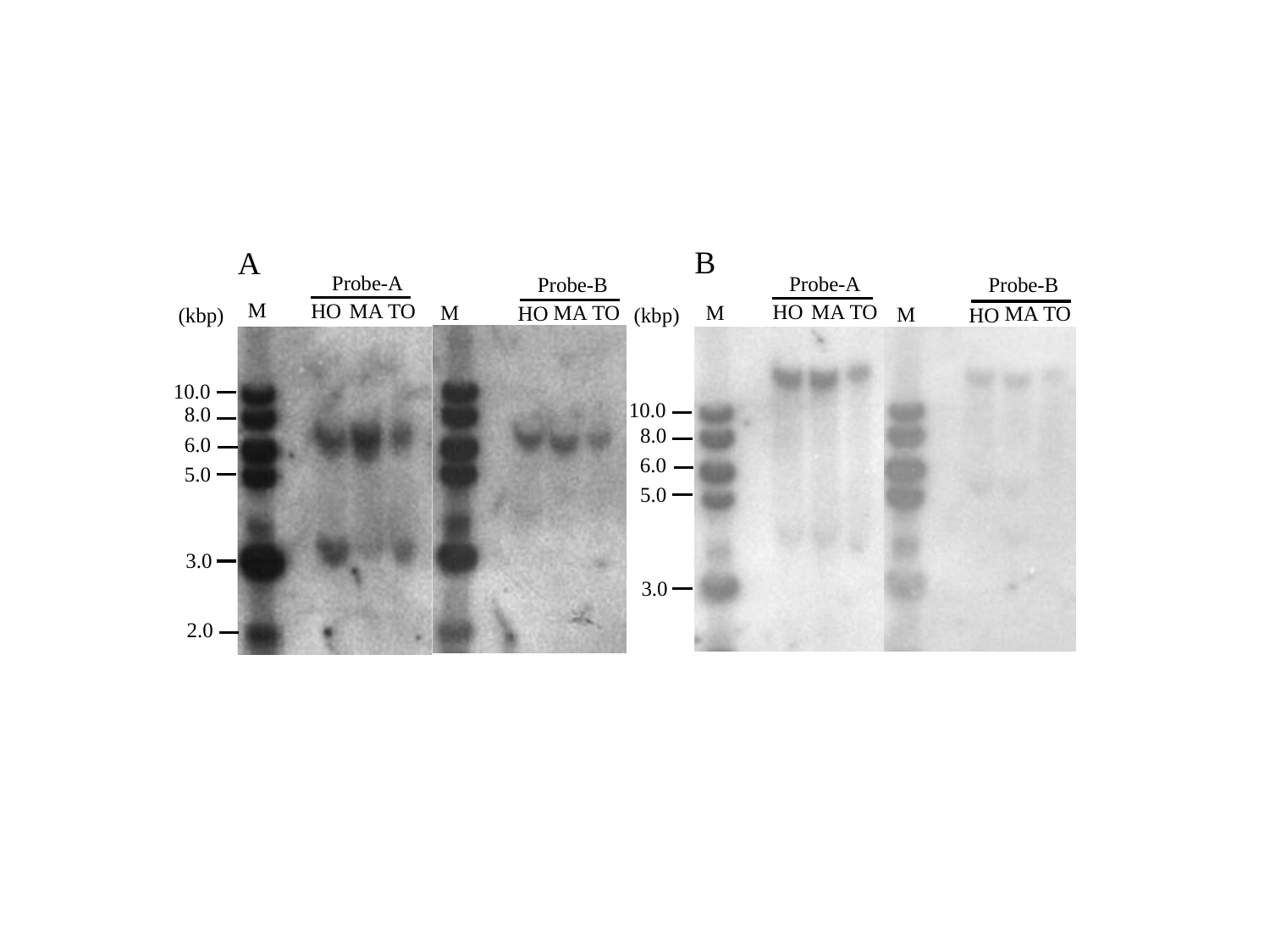

B
A
Probe-A
Probe-A
Probe-B
Probe-B
M
HO
MA
TO
HO
MA
TO
MA
TO
M
M
HO
MA
TO
M
HO
(kbp)
(kbp)
10.0
10.0
8.0
8.0
6.0
6.0
5.0
5.0
3.0
3.0
2.0

Supplement: Additional file 3: Figure S2 — Southern blot analyses of the FT-like gene, FcFT1, from Ficus carica L. in fig cultivars. Fig genomic DNA was digested with XbaI and HindIII. Two FcFT1 genomic DNA fragments were labeled with 32P and used as probes. Hybridization and washing were performed under highly stringent conditions as described by Brown (2001). Panel A, digestion with XbaI. Panel B, digestion with HindIII. Fig cultivars used were as follows: HO, ‘Houraishi’; MA, ‘Masui Dauphine’; TO, ‘Toyomitsuhime’; M, 1 kb ladder marker. Accession numbers: probe-A (AB594722), probe-B (AB594723). [file 1471-2229-13-216-S3.pptx]

## Slide 1
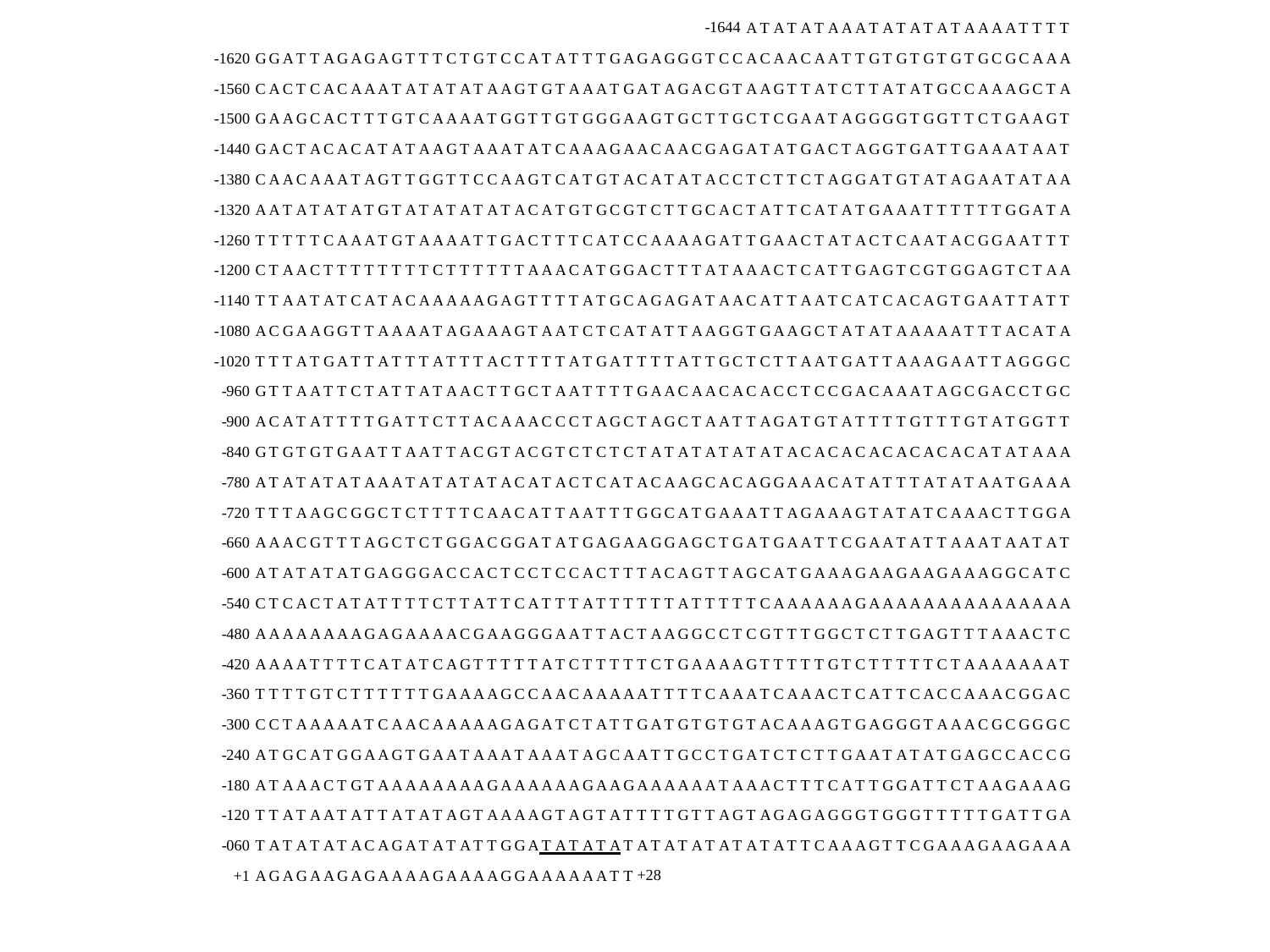

Supplement: Additional file 4: Figure S3 — Promoter sequence of the FT-like gene, FcFT1, from Ficus carica L. The 1,644-bp genomic DNA fragment flanking the 5′ end of the gene contains several putative regulatory elements including an underlined TATA-box. [file 1471-2229-13-216-S4.pptx]
