# Supplementary material for: Expression of FcFT1, a FLOWERING LOCUS T-like gene, is regulated by light and associated with inflorescence differentiation in fig (Ficus carica L.)
Source: BMC Plant Biol. 2013 Dec 16;13:216. doi: 10.1186/1471-2229-13-216 (PMC3878838; doi:10.1186/1471-2229-13-216)
Supplement: Additional file 5: Table S2 — Cis-element sequences identified in the 5′ upstream region of the FT-like gene FcFT1, from Ficus carica L. [file 1471-2229-13-216-S5.doc]

**Additional file 5: Table S2** Cis-element sequences identified in the 5' upstream region of the *FT*-like gene *FcFT1* in *Ficus carica* L
